# Comparative diagnostic accuracy of the triglyceride/HDL-c ratio and lipid accumulation product index for the early detection of metabolic syndrome among adults with obesity in Indonesia: findings from the 2023 Health Survey

**DOI:** 10.20945/2359-4292-2026-0016

**Published:** 2026-02-16

**Authors:** Alya Ayu Alvitananda, Etika Ratna Noer, Ahmad Syauqy, Edward Kurnia Setiawan Limijadi, Adriyan Pramono

**Affiliations:** 1 Department of Nutrition, Faculty of Medicine, Diponegoro University, Semarang, Indonesia; 2 Department of Clinical Pathology, Faculty of Medicine, Diponegoro University, Semarang, Indonesia

**Keywords:** Metabolic syndrome, obesity, predictor

## Abstract

**Objective:**

To evaluate the diagnostic performance and determine the optimal cutoff values of the triglyceride-to-high-density lipoprotein cholesterol ratio and lipid accumulation product index as predictors of metabolic syndrome among adults with obesity in Indonesia.

**Materials and methods:**

This cross-sectional study analyzed secondary data from the 2023 Indonesia Health Survey, which included 3,988 samples (2,958 women). Descriptive statistics were used to characterize the sample. Receiver Operating Characteristic curve analysis and the Youden index were employed to assess diagnostic performance and determine the optimal cutoff values of the triglyceride-to-high-density lipoprotein cholesterol ratio and lipid accumulation product index. The associations between both predictors and the presence of metabolic syndrome were examined using multivariable logistic regression.

**Results:**

The lipid accumulation product index exhibited greater predictive accuracy than the triglyceride-to-high-density lipoprotein cholesterol ratio, particularly among men. This result indicated the superior utility of the lipid accumulation product index as a clinical screening tool for metabolic syndrome, with area under the curve values of 0.842 (95% CI 0.817-0.866) for men and 0.737 (95% CI 0.720-0.755) for women, compared to that of the triglyceride-to-high-density lipoprotein cholesterol ratio, with area under the curve values of 0.810 (95% CI 0.784–0.837) for men and 0.728 (95% CI 0.710-0.746) for women. The optimal cutoff values of the triglyceride-to-high-density lipoprotein cholesterol ratio and lipid accumulation product index were 4.456 (sensitivity 64.8%, specificity 81.4%) and 45.752 (sensitivity 75.5%, specificity 81.2%) for men and 2.792 (sensitivity 59.2%, specificity 76.8%) and 41.285 (sensitivity 58.6%, specificity 75.8%) for women, respectively.

**Conclusion:**

The lipid accumulation product index demonstrated superior accuracy in predicting metabolic syndrome among adults with obesity, particularly among men. Sex-specific cutoff values enhance its reliability and practicality for early screening and intervention to prevent metabolic complications.

## INTRODUCTION

Metabolic syndrome (MetS) is a complex condition characterized by a cluster of metabolic disorders, including central obesity, dyslipidemia, hypertension, and insulin resistance (IR) ^(1,2)^. Metabolic syndrome is associated with increased risks of cardiovascular disease (CVD), stroke, and type 2 diabetes mellitus (T2DM) and contributes significantly to global morbidity and mortality ^(3,4)^. The global prevalence of MetS is increasing, particularly in developing countries, primarily because of increasing obesity rates among adults ^(5)^. In the Asia-Pacific region, nearly one-fifth or more of the adult population in many countries has been diagnosed with MetS, exhibiting an increasing trend ^(6)^.

In Indonesia, the prevalence of MetS is estimated at 21.66%, with a disproportionately higher rate among women (46%), which is nearly twice that among men (28%) ^(7)^. This high prevalence is accompanied by an 8.6% increase in obesity over the past decade ^(8-10)^. Obesity, a major public health concern, is a key contributor to MetS through mechanisms such as ectopic fat accumulation and systemic inflammation, which promote IR, a central pathway in the development of metabolic and physiological disturbances associated with MetS ^(11,12)^. As a result, this condition significantly increases the risk of cardiometabolic complications and contributes to national morbidity and mortality ^(13)^.

Although several international organizations have proposed diagnostic criteria for MetS ^(14-17)^, inclu­ding the widely recognized Harmonizing the Metabolic Syndrome: A Joint Interim Statement 2009 ^(18)^, their implementation in large-scale population screenings remains challenging in Indonesia. These challenges are particularly evident in primary health care settings, where limited resources and uneven health care infrastructure pose significant barriers. In addition, comprehensive and up-to-date data on the prevalence of MetS at national, regional, and population-specific levels remain scarce ^(19)^. Therefore, there is a need for simpler and more accessible screening tools to support the early detection and prevention of MetS.

Given that fat mass and its distribution are significant metabolic factors, various cost-effective and simple alternative biomarkers have been explored to improve the accuracy of MetS diagnosis. Among them are the triglyceride-to-high-density lipoprotein cholesterol ratio (TG/HDL-c) and the lipid accumulation product (LAP) index ^(20-22)^. TG/HDL-c, which integrates key lipid components, has shown potential as a surrogate marker for MetS, IR, and atherosclerosis severity ^(23,24)^. Similarly, the LAP index combines waist circumference (WC) and triglyceride (TG) levels, providing a practical measure of visceral fat accumulation ^(25)^.

The LAP index and TG/HDL-c, which incorporate WC and lipid parameters, not only reflect underlying metabolic dysfunction but also offer more accurate assessment of visceral adiposity and metabolic risk. In this study, both markers demonstrated superior diagnostic performance for MetS, as indicated by their higher AUC values. In contrast, conventional anthropometric indicators such as body mass index (BMI), WC, waist-to-hip ratio (WHR), and waist-to-height ratio (WHtR), while easy to measure, present notable limitations. Body mass index is widely used to assess general obesity, but it does not differentiate between muscle mass and body fat. Similarly, waist-based indicators such as WC, WHR, and WHtR can reflect central obesity but are unable to distinguish between visceral and subcutaneous fat ^(22,26)^.

Although useful, the diagnostic performance and optimal cutoff values of the TG/HDL-c and LAP index are influenced by several factors, including population characteristics, ethnicity, sex, geographic region, and lifestyle habits such as diet, physical activity, and medication use ^(27)^. These variations underscore the importance of context-specific validation, particularly in diverse populations such as those inhabiting Indonesia. Nonetheless, their accessibility and ease of use make these markers appealing for early MetS screening and risk stratification ^(28)^.

To date, no study has systematically compared the diagnostic performance and determined the optimal cutoff values of the TG/HDL-c ratio and LAP index among adults with obesity in the Indonesian population. Therefore, this study aims to evaluate and compare the effectiveness of the TG/HDL-c ratio and LAP index as predictors of MetS and to determine their optimal cutoff values for this specific population.

## MATERIALS AND METHODS

This study received ethical approval from Pusat Data dan Teknologi Informasi (PUSDATIN) Kementerian Kesehatan Republik Indonesia (Approval Number: FRM/SMKI-PUSDATIN/70/0226/2024) and the Ethics Committee of Diponegoro University Faculty of Medicine (Approval Number: No. 469/EC/KEPK/FK-UNDIP/IX/2024).

### Study population

This study employed a cross-sectional design using secondary data from the 2023 Indonesian Health Survey (IHS), which encompassed 345,000 households across 34,500 census blocks in 514 districts or cities from 38 provinces in Indonesia. To ensure data quality, the IHS incorporated interagency cooperation, pilot studies, enumerator training, technical supervision, external validation, quality control, and tool calibration. Stratification was performed both explicitly at the census block level (based on area classification and access to health care) and implicitly at the household level (based on the education of the head of household). Census blocks were selected using the probability proportional to size (PPS) method to ensure proportional regional representation, and households were selected systematically. National biomedical estimates were derived from 2,500 census blocks with individual selection to ensure population representativeness ^(10)^.

From this subsample, subjects were selected via total sampling, including those who met the inclusion criteria: age 19 to 64 years; having obesity, defined as a BMI of ≥ 25 kg/m²; and complete data on demographic characteristics, lifestyle (i.e., smoking and physical activity), lipid profile, fasting blood glucose (FBG), BMI, blood pressure, and WC are available. Subjects with incomplete (missing) data were excluded using the listwise deletion method. Individuals with extreme values were also excluded based on predefined criteria. Extreme data were defined as those with skewness or kurtosis values outside the range of -1 to +1 and identified as outliers on the basis of stem-and-leaf plots ^(29)^. The final sample size analyzed in this study was 3,988 individuals.

### Basic characteristics

All the data in this study were secondary data previously collected by the IHS Team in 2023. The demographic and lifestyle characteristics included age, sex, place of residence, education, physical activity, and smoking behavior. This data was gathered through interviews by using a validated, structured questionnaire. The physical activity and smoking questionnaires were tested and validated by the IHS Team to ensure the accuracy of question flow, content, and implementation in the Indonesian population.

Physical activity levels were assessed using the Global Physical Activity Questionnaire (GPAQ), which is part of the WHO STEPwise approach to NCD risk factor surveillance (STEPS) program. Research conducted in nine countries, including Indonesia, has highlighted the good reliability and validity of the GPAQ, confirming its suitability for monitoring physical activity in the Indonesian population ^(10,30,31)^.

Physical activity behaviors were categorized into two groups: sufficient (≥ 150 minutes per week of combined vigorous and moderate activity) and insufficient (< 150 minutes per week of combined activity). Smoking status was classified as follows: ever smokers (those who smoked daily or occasionally in the past month or those with a history of smoking) and nonsmokers (those who had never smoked up to the time of data collection). Education level was categorized into two groups: high (≥ high school) and low (< high school) ^(10,30)^.

### Anthropometric measurements

The anthropometric data collected in this study included weight, height, and WC. All measurements were conducted by trained health care personnel following standardized procedures. Instruments were calibrated to ensure accuracy and consistency. Weight was measured using a digital scale (0.1 kg accuracy), while height was measured using a stadiometer (1 mm accuracy). Body mass index was calculated using the formula weight (kg)/height (m²), and a BMI of ≥ 25 kg/m² was classified as obesity. Waist circumference was measured at the midpoint between the lowest rib and the top of the pelvic bone, with the tape wrapped around the body passing through the umbilicus. This measurement was used to assess central obesity. Blood pressure was measured twice on the left upper arm using a digital sphygmomanometer ^(10)^.

### Biochemical data

The biochemical data analyzed in this study included FBG, TG, and high-density lipoprotein (HDL) levels. Blood samples were collected by trained medical personnel who had undergone standardized training. Samples were collected after the subjects had fasted for 8 to 12 hours. Fasting blood glucose was measured using capillary blood samples analyzed with the Accu-Chek Performa device. TG and HDL levels were measured using venous blood samples analyzed by a chemical autoanalyzer with enzymatic methods ^(10)^.

A 7 mL-venous blood sample was drawn and placed in a yellow tube for biochemical analysis. After centrifugation at 3,000 rpm for 10 minutes, the serum was transferred to 5 mL microtubes and stored at -20 °C. The samples were transported to the National Health Laboratory within 48 hours using standardized packaging and shipping protocols. Potential confounders, such as chronic illnesses, bleeding disorders, anticoagulant use, and other physician-identified conditions, were considered during collection ^(10)^.

### Definition of metabolic syndrome

Metabolic syndrome was the dependent variable in this study and was diagnosed using the Harmonizing the Metabolic Syndrome: A Joint Interim Statement 2009 criteria, which had previously been applied in prevalence studies of MetS across various provinces and ethnic groups in Indonesia. The WC cutoff was adjusted explicitly for the Indonesian population ^(32)^. Subjects were classified as having MetS if they exhibited at least three of the following five risk factors: (1) central obesity: WC > 80 cm in women and > 90 cm in men; (2) high TG: ≥ 150 mg/dL (1.7 mmol/L); (3) low HDL: HDL < 40 mg/dL (1.03 mmol/L) in men or < 50 mg/dL (1.29 mmol/L) in women; (4) high blood pressure: systolic blood pressure ≥ 130 mmHg or diastolic ≥ 85 mmHg; and (5) high FBG: blood glucose ≥ 100 mg/dL (5.6 mmol/L) ^(18)^.

### Triglyceride/HDL-c and lipid accumulation product index

The TG/HDL-c and LAP index were calculated using the following formulas:

TG/HDL-c formula: TG (mg/dL)/HDL (mg/dL) ^(4)^

LAP index formula:

Men = WC (cm) – 65 x TG (mmol/L) ^(33)^

Women = WC (cm) – 58 x TG (mmol/L) ^(33)^

Since the original LAP index formula utilizes TG values in mmol/L, TG concentrations initially recorded in mg/dL were converted to mmol/L by dividing by 88.57 prior to calculation.

### Statistical analysis

Data was analyzed using Statistical Package for the Social Sciences (SPSS) version 26.0 for Windows and Microsoft Excel and MedCalc, with statistical significance set at p < 0.05. Numerical variables are presented as the means and standard deviations, whereas categorical variables are presented as counts and percentages.

Data normality, skewness, and kurtosis values were evaluated using stem-and-leaf plots. Data were considered normally distributed if the skewness and kurtosis values fell within the range of -1 to +1 and if no extreme outliers were identified ^(29)^.

Independent *t* tests were used to compare numerical variables, whereas Chi-squared tests were used to compare categorical variables between men and women. Diagnostic accuracy was assessed using the area under the curve (AUC) analysis of the Receiver Operating Characteristic (ROC) curve, stratified by sex. The indicator with the highest AUC was considered the best predictor, with values closer to 1 indicating better accuracy. The AUC values were classified as follows: very weak (0.5 to 0.6), weak (0.6 to 0.7), moderate (0.7 to 0.8), good (0.8 to 0.9), and excellent (> 0.9) ^(34)^.

To statistically compare the AUCs of the TG/HDL-c ratio and the LAP index, DeLong’s test for two correlated ROC curves was employed. This method allowed assessment of whether the discriminative ability of one marker was significantly superior to that of the other, with analyses performed separately for men and women.

The optimal cutoff values of the TG/HDL-c and LAP index were determined using the following Youden index equation: Youden index = sensitivity + specificity -1 ^(35)^.

Finally, a logistic regression model using the full model approach was employed to assess the associations between the TG/HDL-c, the LAP index, and the presence of MetS. The analysis adjusted for potential confounders, including age, sex, BMI, education level, place of residence, smoking status, and physical activity, to ensure more accurate and reliable results.

## RESULTS

**Figure 1:**
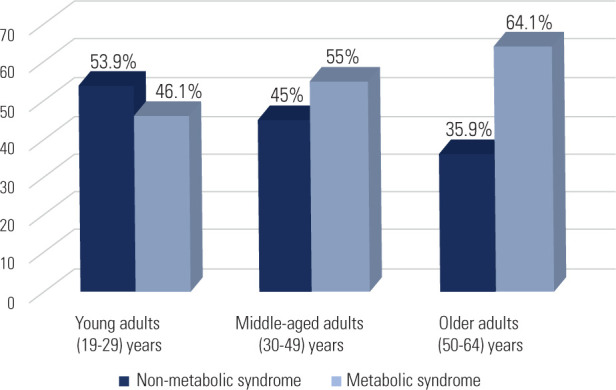
Prevalence of metabolic syndrome across adult age groups.

This study analyzed 3,988 samples, comprising 1,030 men and 2,958 women aged 19 to 64. The prevalence of MetS significantly increased with age, as illustrated in **Figure 1**. The overall prevalence of MetS in this study population was 56%, with a slightly higher prevalence observed in men (56.6%) than in women (55.7%), although this difference was not statistically significant (p > 0.05) (**Table 1**).

**Table 1.** Characteristics of participants stratified by sex

Variables	Men (1,030) N%	Women (2,958) N%	Total (3,988) N%	p-value
Prevalence of metabolic syndrome				
Non-metabolic syndrome	447 (43.4)	1,309 (44.3)	1,756 (44)	0.634
Metabolic syndrome	583 (56.6)	1,649 (55.7)	2,232 (56)	
Age, years	43.49 ± 11.35	40.90 ± 10.25	41.57 ± 10.61	< 0.001*
Body mass index, kg/m^2^	27.74 ± 2.42	28.85 ± 3.25	28.56 ± 3.09	< 0.001*
Education level				
Low	431 (41.8)	1,695 (42.7)	2126 (53.3)	< 0.001*
High	599 (58.2)	1,263 (57.3)	1,862 (46.7)	
Place of residence				
Rural	351 (34.1)	1,134 (38.3)	1,485 (37.2)	0.015*
Urban	679 (65.9)	1,824 (61.7)	2,503 (62.8)	
Smoking status				
Nonsmoker	422 (41.0)	2,909 (98.3)	3,331 (83.5)	< 0.001*
Ever smoker	608 (59.0)	49 (1.7)	657 (16.5)	
Physical activity				
Low	365 (35.4)	682 (23.1)	1,047 (26.3)	< 0.001*
Moderate	665 (64.6)	2,274 (76.9)	2,939 (73.7)	
Components of metabolic syndrome				
Waist circumference, cm	91.60 ± 7.83	89.35 ± 8.06	89.93 ± 8.06	< 0.001*
Triglyceride level, mg/dL	168.42 ± 66.94	116.09 ± 39.79	129.61 ± 53.44	< 0.001*
HDL level, mg/dL	37.49 ± 6.65	43.85 ± 8.14	42.1 ± 8.27	< 0.001*
Systolic blood pressure, mmHg	128.95 ± 13.32	125.67 ± 15.79	126.52 ± 15.26	< 0.001*
Diastolic blood pressure, mmHg	84.24 ± 8.63	84.71 ± 8.81	84.59 ± 8.76	0.144
Fasting blood glucose level, mg/dL	98.18 ± 8.71	94.96 ± 7.79	95.79 ± 8.16	< 0.001*
Predictors of metabolic syndrome				
TG/HDL-c	4.70 ± 2.16	2.76 ± 1.11	3.26 ± 1.68	< 0.001*
LAP index	51.00 ± 25.59	41.41 ± 18.39	43.89 ± 20.91	< 0.001*

Results expressed as n (%) or mean ± standard deviation.

The p-values were calculated using the Chi-squared test for categorical variables and the independent *t* test for numerical variables.

* Statistical significance was set at p < 0.05.

HDL: high-density lipoprotein; TG/HDL-c: triglyceride-to-high-density lipoprotein cholesterol ratio; LAP: lipid accumulation product.

The characteristics of the participants stratified by sex are presented in **Table 1**. Significant differences were observed between men and women in terms of age, BMI, educational level, place of residence, smoking status, physical activity, WC, triglyceride levels, HDL levels, systolic blood pressure, fasting blood glucose levels, the TG/HDL-c, and the LAP index (p < 0.05). However, no significant difference in diastolic blood pressure was detected (p > 0.05). Additionally, the average age of men (43.49 ± 11.35) was slightly greater than that of women (40.90 ± 10.25).

**Table 2** displays the baseline characteristics of participants with and without MetS, stratified by sex. For men, significant differences (p < 0.05) were found in all the variables except for education, place of residence, smoking status, and physical activity. In women, almost all the variables were significantly different, except place of residence, smoking status, and physical activity (p > 0.05). As expected, most components of MetS, including WC, TG, blood pressure, FBG, the TG/HDL-c, and the LAP index, exhibited worse values in the MetS group, while HDL levels were lower.

**Table 2.** Baseline characteristics of study participants with and without metabolic syndrome, stratified by sex

Variables N%	Men		p-value	Women		p-value
	Without MetS N%	With MetS N%		Without MetS N%	With MetS N%	
Age, years	42.40 ± 11.35	44.32 ± 11.29	0.007*	39.07 ± 10.13	42.35 ± 10.12	< 0.001*
Body mass index, kg/m^2^	27.04 ± 2.00	28.27 ± 2.58	< 0.001*	28.28 ± 3.16	29.30 ± 3.24	< 0.001*
Education level						
Low	181 (40.5)	250 (42.9)	0.441	698 (53.3)	997 (39.5)	< 0.001*
High	266 (59.5)	333 (57.1)		611 (46.7)	652 (60.5)	
Place of residence						
Rural	157 (35.1)	194 (33.3)	0.535	484 (37.0)	650 (39.4)	0.175
Urban	290 (64.9)	389 (66.7)		825 (63.0)	999 (60.6)	
Smoking status						
Nonsmoker	182 (40.7)	240 (41.2)	0.884	1287 (98.3)	1622 (98.4)	0.927
Ever smoker	265 (59.3)	343 (58.8)		22 (1.7)	27 (1.6)	
Physical activity						
Low	149 (33.3)	216 (37.0)	0.217	306 (23.4)	376 (22.8)	0.695
Moderate	298 (66.7)	367 (63.0)		1001 (76.6)	1273 (77.2)	
Components of metabolic syndrome						
Waist circumference, cm	88.03 ± 7.30	94.33 ± 7.08	< 0.001*	86.86 ± 8.41	91.34 ± 7.18	< 0.001*
Triglyceride level, mg/dL	135.05 ± 56.44	194.02 ± 63.02	< 0.001*	100.85 ± 30.78	128.19 ± 41.93	< 0.001*
HDL level, mg/dL	40.38 ± 6.89	35.27 ± 5.51	< 0.001*	46.40 ± 8.69	41.84 ± 7.06	< 0.001*
Systolic blood pressure (mmHg)	124.60 ± 11.79	132.29 ± 13.47	< 0.001*	118.62 ± 13.41	131.26 ± 15.29	< 0.001*
Diastolic blood pressure (mmHg)	81.23 ± 8.00	86.56 ± 8.38	< 0.001*	80.37 ± 7.62	88.15 ± 8.14	< 0.001*
Fasting blood glucose level, mg/dL	94.87 ± 7.35	100.73 ± 8.82	< 0.001*	92.06 ± 6.27	97.26 ± 8.10	< 0.001*
Predictors of metabolic syndrome						
TG/HDL-c	3.46 ± 1.62	5.6 ± 2.05	< 0.001*	2.27 ± 0.85	3.15 ± 1.13	< 0.001*
LAP index	34.69 ± 17.20	63.50 ± 23.87	< 0.001*	32.96 ± 14.23	48.12 ± 18.55	< 0.001*

Results expressed as mean ± standard deviation or n (%).

The p-values were calculated using the Chi-squared test for categorical variables and the independent *t* test for numerical variables.

* Statistical significance was set at p < 0.05.

MetS: matabolic syndrome; HDL: high-density lipoprotein; TG/HDL-c: triglyceride-to-high-density lipoprotein cholesterol ratio; LAP: lipid accumulation product.

**Figure 2:**
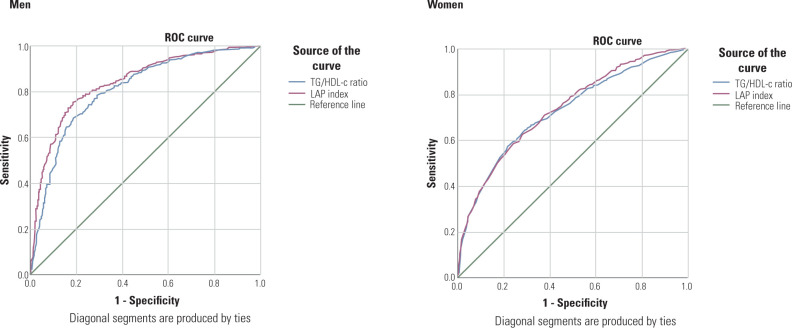
Receiver Operating Characteristic curves of each indicator in the prediction of metabolic syndrome risk based on sex.

ROC: Receiver Operating Characteristic; TG/HDL-c: triglyceride-to-high-density lipoprotein cholesterol ratio; LAP: lipid accumulation product.

**Table 3** and **Figure 2** present the ROC analysis results for the TG/HDL-c and LAP index as predictors of MetS stratified by sex. Both indicators showed good predictive ability for MetS in men (AUC = 0.8-0.9; p < 0.001), with the LAP index demonstrating superior performance (AUC = 0.842; 95% CI 0.817-0.866) compared to the TG/HDL-c (AUC = 0.810; 95% CI 0.784-0.837), and this difference was statistically significant, as determined by DeLong’s test (p < 0.05).

**Table 3.** Outcomes of the Receiver Operating Characteristic curve for men and women

Predictors of metabolic syndrome	AUC	95% CI	Standard error	p-value	Sensitivity (%)	Specificity (%)	Youden index	Optimal cutoff	LR+	LR-
Men										
TG/HDL-c	0.810	0.784-0.837	0.14	< 0.001*	64.8	81.4	0.499	4.456	3.686	0.388
LAP	0.842	0.817-0.866	0.12	< 0.001*	75.5	81.2	0.567	45.752	4.016	0.302
Women										
TG/HDL-c	0.728	0.710-0.746	0.009	< 0.001*	59.2	76.8	0.360	2.793	2.549	0.532
LAP	0.737	0.720-0.755	0.009	< 0.001*	58.6	75.8	0.344	41.285	2.419	0.547

* Statistically significant (p-value < 0.05).

AUC: area under the curve; 95% CI 95% of confidence interval; LR+: likelihood ratio +; LR-: likelihood ratio-; TG/HDL-c: triglyceride-to-high-density lipoprotein cholesterol ratio; LAP: lipid accumulation product.

In women, both indicators showed moderate predictive ability (AUC = 0.7–0.8; *p* < 0.001). Although the LAP index had a slightly greater AUC (0.737; 95% CI 0.720–0.755) than the TG/HDL-c did (AUC = 0.728; 95% CI 0.710-0.746), the difference was not statistically significant (p > 0.05). Detailed results of the AUC comparisons using DeLong’s test are presented in **Supplementary Table 1**.

Furthermore, in this study, the optimal cutoff values were determined using the Youden index. For men, the TG/HDL-c had a Youden index of 0.499, with an optimal cutoff value of 4.456 (Sn = 64.8%, Sp = 81.4%), while the LAP index had a Youden index of 0.567, with an optimal cutoff value of 45.752 (Sn = 75.5%, Sp = 81.2%). In women, the TG/HDL-c had a Youden index of 0.360, with an optimal cutoff value of 2.793 (Sn = 59.2%, Sp = 76.8%), whereas the LAP index had a Youden index of 0.344, with an optimal cutoff value of 41.285 (Sn = 58.6%, Sp = 75.8%).

As shown in **Table 4**, both the TG/HDL-c and the LAP index were independently and significantly associated with the incidence of MetS across all the models (p < 0.001), including the fully adjusted Model 6, which controlled for age, sex, BMI, education level, place of residence, smoking status, and physical activity.

**Table 4.** Associations of the triglyceride-to-high-density lipoprotein cholesterol ratio and lipid accumulation product index with metabolic syndrome: analysis with adjustment for confounding variables

	Model unadjusted			Model adjusted for age and sex			Mode adjusted body mass index		
	OR	95% CI	p-value	OR	95% CI	p-value	OR	95% CI	p-value
TG/HDL-c	1.207	1.129-1.290	< 0.001*	1.479	1.365-1.603	< 0.001*	1.270	1.184-1.364	< 0.001*
LAP	1.049	1.043-1.055	< 0.001*	1.040	1.034-1.046	< 0.001*	1.043	1.037-1.050	< 0.001*

	Model adjusted for education level and place of residence			Model adjusted for smoking status and physical activity			Model adjusted for age, sex, body mass index, education level, place of residence, smoking status, and physical activity		
	OR	95% CI	p-value	OR	95% CI	p-value	OR	95% CI	p-value
TG/HDL-c	1.211	1.133-1.296	< 0.001*	1.332	1.237-1.435	< 0.001*	1.574	1.445-1.714	< 0.001*
LAP	1.049	1.043-1.055	< 0.001*	1.045	1.040-1.051	< 0.001*	1.033	1.027-1.040	< 0.001*

* Significant, p < 0.05.

OR: odds ratio; 95% CI 95% of confidence interval; TG/HDL-c: triglyceride-to-high-density lipoprotein cholesterol ratio; LAP: lipid accumulation product.

In the male subgroup, the LAP index demonstrated a significantly greater AUC than the TG/HDL-c did (p < 0.05). In contrast, among women, the LAP index had a slightly greater AUC than the TG/HDL-c did; however, the difference was not statistically significant (p > 0.05) (**Supplementary Table 2**).

Consistent with the primary findings, external validation using data from the 2018 Indonesian Basic Health Survey (IHS) in a population of adults with obesity (age: 19 to 50 years; BMI ≥ 25 kg/m²; *n* = 4,727, comprising 999 men and 3,728 women) confirmed moderate to good discriminative ability of both the TG/HDL-c and LAP index in predicting MetS. The LAP index consistently demonstrated better performance than the TG/HDL-c in men, although the difference was not statistically significant.

## DISCUSSION

In this study, both the TG/HDL-c and LAP index were found to be useful for predicting MetS, with the LAP index showing better performance, especially in adult men with obesity. The analysis employed an obesity cutoff of BMI ≥ 25 kg/m², which is more appropriate for Asian populations, including the Indonesian population, and aligns with current recommendations to enhance the clinical evaluation and prevention of metabolic complications ^(36)^. The LAP index is a key obesity indicator that reflects visceral fat accumulation on the basis of the WC component and atherogenic dyslipidemia on the basis of triglyceride levels ^(12,22)^.

Visceral fat has higher metabolic and proinflammatory activity than subcutaneous fat does ^(37)^. This fat produces free fatty acids (FFA) and proinflammatory cytokines and decreases adiponectin, which triggers IR, dyslipidemia, renin-angiotensin-aldosterone system (RAAS) activation, and hyperglycemia ^(25,38,39)^. Moreover, atherogenic dyslipidemia occurs when the liver increases the production of very low-density lipoprotein (VLDL) in response to excessive lipolysis of FFAs due to IR. This lipid accumulation exacerbates IR through lipotoxicity, resulting in the formation of a negative feedback loop. Therefore, the LAP index provides a comprehensive overview of cardiometabolic risk in MetS ^(40)^.

A study from China evaluating obesity-related parameters and lipids to predict MetS revealed that the LAP index was superior to other measures, such as the Visceral Adiposity Index (VAI), TG/HDL-c, WHtR, and BMI. In line with our research, the LAP index performed better than the TG/HDL-c ^(41)^.

In this study, the LAP index demonstrated a strong ability to predict MetS, particularly in men with adequate Sn values and high Sp-values. In women, the performance of this index was moderately effective, but still clinically relevant. These findings are consistent with those of previous cohort studies in China that evaluated 13 indices related to obesity and lipids and revealed that the LAP index has excellent predictive ability in men (AUC = 0.912; 95% CI 0.903-0.921), with a cutoff of 27.895 (Sn = 83.5%, Sp = 83.6%), whereas in women, the LAP index has a slightly lower AUC of 0.876 ( 95% CI 0.867–0.885), with a cutoff of 35.867 (Sn = 73.2%, Sp = 83.4%) ^(42)^.

The high consistency of the AUC values of the LAP index in various international studies supports its reliability as a tool for identifying MetS risk ^(43,44)^. The superior performance of the LAP index in men in this study was also assessed, with a Sp-value above 80%, supporting its use in early screening to minimize overdiagnosis and improve targeted interventions ^(45)^.

Moreover, the TG/HDL-c may better reflect atherogenic dyslipidemia, but this ratio does not give sufficient importance to body fat distribution, which makes it less specific for assessing MetS risk, especially in populations with obesity. This ratio component, especially the HDL, is influenced by external factors and fluctuates. HDL-c dysfunction can also cause high HDL levels despite increased metabolic risk, thereby reducing Sn levels and the AUC ^(46)^. Therefore, the TG/HDL-c is more appropriately considered a systemic biomarker ^(47)^.

However, the TG/HDL-c also has good predictive ability, although slightly less than the LAP index, particularly in women. As further support, a study in Iran revealed that the TG/HDL-c is a potential indicator of MetS (AUC = 0.85 in both men and women), which is consistent with our findings, although the AUC values in that study were slightly greater ^(48)^. Similarly, a study of elderly individuals in China revealed that the TG/HDL-c demonstrated good predictive ability (AUC = 0.813; 95% CI 0.784-0.842), with slightly lower cutoff values: 1.437 for men (Sn = 74.8% and Sp = 78.4%) and 1.196 for women (Sn = 86.4% and Sp = 67.4%) ^(49)^.

Notably, the differences in the AUC and cutoff values between our study and others may be attributed to variations in genetic background, lifestyle factors, body composition, dietary habits, and the use of different MetS criteria across populations.

This study also highlights significant differences in LAP index values and TG/HDL-c between sexes, with men having higher average values. These findings are consistent with the characteristics of the components of both indicators, such as WC and lipid levels. Men have higher WC and TG levels and lower HDL levels, whereas women generally have normal TG levels and higher HDL levels. This combination results in higher final values for both predictors, indicating a higher risk for men.

These observations are closely related to body fat distribution, which is influenced by hormonal and physiological factors. Men tend to store fat in the visceral area, leading to a higher WC, even though the BMI was higher in women in this study. This visceral fat distribution is influenced by testosterone. Women store more fat in the subcutaneous area, particularly in the thighs and hips, because of the influence of estrogen. Subcutaneous fat does not significantly increase WC, despite having a higher total fat mass ^(50)^.

Visceral fat increases the release of FFA and TG synthesis in the liver, whereas estrogen increases lipoprotein lipase (LPL) activity, which accelerates TG metabolism ^(51)^. These findings indicate that men are more likely to experience metabolic disorders ^(52)^. Moreover, lower HDL levels in men are also associated with higher liver lipase activity ^(53)^.

In addition, men tend to have higher systolic blood pressure and FBG levels because of the influence of testosterone, which increases RAAS activity. Conversely, estrogen in women protects blood vessels by lowering RAAS, acting as an anti-inflammatory agent, and maintaining HDL levels. ^(54)^. Moreover, there were no significant differences in diastolic blood pressure, possibly because vascular compensation maintains its stability ^(55)^.

The aforementioned explanation elucidates the rationale behind the superior predictive ability of the LAP index compared to the TG/HDL-c in men, with both indices demonstrating greater predictive power than those in the female cohort. This disparity is likely attributable to the influence of testosterone, a more pronounced accumulation of visceral fat, and more significant dyslipidemia in men. Conversely, in women, the predominance of subcutaneous fat distribution, more stable triglyceride levels, and elevated HDL levels may contribute to the lower predictive performance values relative to men. The corresponding predictive abilities of the LAP index and the TG/HDL-c were comparable, with no significant differences observed between the two. Furthermore, the larger sample size of women, coupled with narrower variability in lipid and anthropometric profiles, may also account for the absence of statistically significant differences identified.

Moreover, lifestyle factors such as smoking and a lack of physical activity also play a role. Our results revealed that compared with women, men ever smoked more and were less active, which increased the risk of MetS. Physical activity increases insulin sensitivity and reduces inflammation, whereas smoking exacerbates metabolic disorders ^(56,57)^. In addition, the older average age of the men in this study was also associated with increased risk of MetS components ^(58)^.

In line with findings that men have more MetS components and riskier lifestyles, the prevalence of MetS in this study was slightly higher in men (56.6%) than in women (55.7%), differing from previous findings that showed a higher prevalence in women ^(59)^. This difference may also be influenced by the larger number of women (n = 2,958) than men (n = 1,030). Although MetS is generally more common in women, men tend to have more severe metabolic disorders ^(60)^.

The prevalence of MetS was 56% higher in this study than in previous studies in Indonesia ^(32)^. This increase, along with high obesity rates, highlights the importance of deploying more effective screening tools for diverse populations so that interventions such as diet management, pharmacological therapy, and lifestyle modifications can be targeted appropriately to prevent MetS complications.

In conclusion, the findings of this study suggest that both the lipid accumulation product index and triglycerides/HDL-c serve as sex-specific predictors of metabolic syndrome in adults with obesity, each with distinct optimal cutoff values. The lipid accumulation product index exhibited superior performance in men and is recommended as a practical screening tool in primary health care and public health contexts in Indonesia, particularly where resources are constrained. In women, both indices demonstrated comparable predictive capabilities; however, the lipid accumulation product index is preferable because of its ease of calculation, practicality, and cost-effectiveness, rendering it more suitable for screening within the general population.

This study contributes to the national literature by using 2023 Indonesian Basic Health Survey data, which are representative of the broader population, and involved trained enumerators to minimize bias. However, this study has several limitations, including its cross-sectional design, which precludes causal inferences. Additionally, the potential for residual confounding remains due to unmeasured variables such as dietary intake, medication use, comorbidities, psychological stress, and genetic predispositions, all of which may influence metabolic outcomes and their interaction with the two predictors, as well as the overall prevalence of metabolic syndrome.

Further research is needed, including longitudinal studies controlling for these factors and more comprehensive clinical and genetic profiles, both in Indonesia and globally. Direct measurement of body fat or visceral fat would also strengthen the findings and provide a more accurate understanding of the role of obesity in metabolic syndrome.

## Data Availability

the data used in this study were obtained from Pusat Data dan Teknologi Informasi (PUSDATIN) of the Kementerian Kesehatan Republik Indonesia through a structured data request and ethical approval.

## Appendix

**Supplementary Table 1.** Area under the curve comparison between the lipid accumulation product index and the triglyceride-to-high-density lipoprotein cholesterol ratio using DeLong’s test stratified by sex

Group	AUC LAP (95% CI)	AUC TG/HDL-c (95% CI)	AUC Difference	95% CI of Difference	p-value (DeLong)
Men	0.842 (0.818-0.863)	0.810 (0.785-0.834)	0.0313	0.00987-0.0527	0.0042*
Women	0.737 (0.721-0.753)	0.728 (0.711-0.744)	0.0095	-0.00531-0.0242	0.2092

* Statistically significant (p-value < 0.05).

AUC: area under the curve; LAP: lipid accumulation product; 95% CI 95% of confidence interval; TG/HDL-c: triglycerides to high-density lipoprotein cholesterol ratio.

**Supplementary Table 2.** External validation of the predictive accuracy of the triglycerides to high-density lipoprotein cholesterol ratio and lipid accumulation product index for MetS in adults with obesity (Indonesian Basic Health Survey 2018 Data)

Predictors of metabolic syndrome	AUC	95% CI	Standard error	p-value	Sensitivity (%)	Specificity (%)	Youden index	Optimal cutoff	LR+	LR-	p-value DeLong Test
Men (n: 999)											
TG/HDL-c	0.825	0.799-0.851	0.13	< 0.001*	81.5	71.5	0.530	3.225	2.862	0.258	0.176
LAP	0.838	0.814-0.863	0.12	< 0.001*	77.6	76.1	0.537	42.150	3.247	0.295	
Women (n: 3728)											
TG/HDL-c	0.766	0.751-0.782	0.008	< 0.001*	59.2	81.0	0.402	2.374	3.119	0.504	0.168
LAP	0.759	0.744-0.774	0.008	< 0.001*	66.2	73.1	0.070	33.737	2.460	0.463	

* Statistically significant (p-value < 0.05),

AUC: area under the curve; 95% CI 95% of confidence interval; LR+: likelihood ratio +; LR-: likelihood ratio -; TG/HDL-c: triglycerides to high-density lipoprotein cholesterol ratiol; LAP: lipid accumulation product.
